# SGLT-2 inhibitors may increase ultrafiltration in incident peritoneal dialysis patients: a case report

**DOI:** 10.1186/s12882-023-03164-8

**Published:** 2023-04-22

**Authors:** Jia-Wen Lai, Hsuan-Jen Lin, Che-Yi Chou

**Affiliations:** 1 Division of Nephrology, Asia University Hospital, Wufeng, Taichung, Taiwan; 2 Division of Nephrology, China Medical University Hospital, Taichung, Taiwan; 3 Department of Post-Baccalaureate Veterinary Medicine, Asia University, NO 222, Fuxin Rd, Wufeng Dist, Taichung 413, Taiwan

**Keywords:** peritoneal dialysis, Ultrafiltration, SGLT-2 inhibitors, Case report

## Abstract

**Background:**

Adequate fluid removal to achieve euvolemic status can be difficult in patients with incident peritoneal dialysis (PD). Limited treatments such as increased high dextrose PD solutions and icodextrin are currently available. We reported four incident PD patients whose’ ultrafiltration volume was increased after sodium-glucose cotransporter-2 inhibitors.

Case presentation.

The four reported cases were diabetic kidney disease stage 5 (cases 1–3) and IgA nephritis (case 4) patients whostartedt PD because of acute pulmonary edema (case 1 and 3), nausea vomiting (case 2), and hyperkalemia (case 4). They had an ultrafiltration volume of 700-1000 ml per day but hpersistentted peripheral pitting edema or pulmonary edema. Their ultrafiltration volincreased after dapagliflozin 5 mg daily, and the fluid overload symptoms ere improved. No hypotension, or hypoglycemia was found, and the urine was not increased during dapagliflozin treatment.

**Conclusions:**

SGLT-2 inhibitors may increase ultrafiltration in incident PD patients. More studies are needed to support the safety of SGLT-2 inhibitors in PD patients.

## Background

In incident peritoneal dialysis (PD) patients, adequate fluid removal can be challenging. Automated PD with short dwelling time, increased exchanges, a higher dextrose concentration, and icodextrin may increase ultrafiltration. Icodextrin usually needs a longer dwelling time and may not increase daily ultrafiltration volume. Sodium-glucose cotransporter-2 (SGLT-2) inhibitors are widely used in the clinic to reduce blood glucose levels by enhancing glucose excretion in the urine. SGLT-2 inhibitors may decrease glucose absorption from PD solution [1] by inhibiting SGLT-2 on the peritoneum. Ultrafiltration may be increased because the glucose in the PD solution may last longer and provide more extended osmotic water transport in PD. SGLT-2 inhibitors may also reduce peritoneal fibrosis by inhibiting the transforming growth factor in animal models [2]. We reported four incident PD cases, and their ultrafiltration was increased after SGLT-2 inhibitors.

## Case presentation

### Case 1

A 52 years old man with diabetic kidney disease started APD because of acute pulmonary edema. His blood pressure is high (182/110 mmHg), and grade 4 pitting edema (the pressure leaves an indentation of 8 mm or deeper, and the indentation takes more than 20 s to rebound) on the lower extremities. The blood urea nitrogen was 175 mg/dl, serum creatinine 16.5 mg/dl, hemoglobin 9.6 g/dl, calcium 8.9 mg/dl, phosphate 6.2 mg/dl, and sugar 185 mg/dl. The PD prescription was one liter, 1-h dwell, eight exchanges, using 2.5% dextrose on the day of PD catheter insertion, followed by two liters, 2-h dwelling on the next day. The average ultrafiltration was 920 ± 257 ml on the first three days, and his acute pulmonary edema was improved. Dapagliflozin, 5 mg per day, was used because the leg pitting edema persisted. The ultrafiltration volume increased to 1260 ± 156 ml, and the urine was 640 ± 180 ml after dapagliflozin. His body weight was 72 kg on discharge. The patient took dapagliflozin was 14 days. Detailed treatment and laboratory data are shown in Table 1.

**Table 1:** Characteristics of four patients

	Case 1	Case 2	Case 3	Case 4
Age year	52	48	57	42
Gender	Male	Female	Male	Female
Blood pressure mmHg	182/110	148/92	174/105	165/92
Height cm	152	165	168	158
Weight kg	82	74	103	65
Body mass index kg/m^2^	35.5	26.1	36.5	26.0
Uremic symptoms	Acute pulmonary edema	Nausea, vomiting	Acute pulmonary edema	Hyperkalemia
Cause of CKD	Diabetes	Diabetes	Diabetes	IgA nephritis
Comorbidity	CAD, CHF, dyslipidemia	-	CAD	-
Before/After				
Venous pH	7.12/7.32	7.18/7.28	7.20/7.35	7.11/7.33
Hemoglobin g/dl	9.6/10.2	8.7/9.8	10.2/10.8	9.2/10/6
BUN mg/dl	175/98	102/85	140/92	172/87
Creatinine mg/dl	16.5/10.2	14.2/9.8	15.8/9.2	18.4/9.5
Calcium mg/dl	8.9/8.9	8.7/8.6	8.5/8.7	7.9/8.2
Phosphate mg/dl	6.5/5.8	3.7/4.3	6.8/5.2	3.8/4.5
Sodium meq/L	142/141	141/141	138/139	137/136
Potassium meq/L	6.2/4.8	4.5/4.2	6.5/3.8	7.2/5.4
Glucose mg/dl	185/162	178/152	194/135	102/98
Modality	Automated	Automated	Automated	CCPD
PET				
D/P	0.96	0.88	0.94	0.86
D/D_0_	0.18	0.24	0.22	0.19
Dialysate sodium meq/L	127	128	125	126
Follow-up days	15	5	8	5
Anti-diabetic drug	Lantus 16u qd	Linagliptin 1# qd	Lantus 20u qd	
Ultrafiltration ml/day				
Basal	920 ± 257	740 ± 322	1142 ± 236	854 ± 385
After SGLT2	1260 ± 156	1140 ± 245	1485 ± 220	1267 ± 288
Urine ml/day				
Basal	580 ± 220	1200 ± 480	220 ± 120	780 ± 530
After SGLT2	640 ± 180	1120 ± 350	320 ± 150	850 ± 250
Weight on discharge kg	72	68	87	59

*CKD* Chronic kidney disease, *CAD* Coronary-artery disease, *CHF* Congestive heart failure, *BUN* Blood urea nitrogen, *PET* Peritoneal equilibration test, *CAPD* Continuous ambulatory peritoneal dialysis, *CCPD* Continuous cycling peritoneal dialysis, *SGLT-2* Sodium-glucose cotransporter-2

### Case 2

A 48 years lady with diabetic kidney disease started automated PD because of nausea, vomiting. Her hemoglobin was 8.7 g/dl, blood urea nitrogen 102 mg/dl, creatinine 14.2 mg/dl, and glucose 178 mg/dl. She was on automated PD with 1 L, 1-h dwell, ten exchanges using 2.5% dextrose after PD catheter placement. Her peritoneum permeability was also high. The ultrafiltration was 740 ± 322 ml on the first three days, and she had mild dyspnea with pulmonary congestion on chest X-ray. The ultrafiltration increased to 1140 ± 245 ml after dapagliflozin, which improved her pulmonary congestion. The patient took dapagliflozin for 28 days.

### Case 3

A 57 years man with hypertension, diabetic kidney disease was on automated PD because of acute pulmonary edema with dyspnea on exertion. The ultrafiltration was 1142 ± 236 ml with 1 L, 1-h dwell, ten exchanges of 2.5% dextrose on the first two days after PD catheter insertion. Dapagliflozin 5 mg per day was prescribed because his dyspnea was not improved. The ultrafiltration volume increased to 1485 ± 220 ml, and his dyspnea was improved. The patient took dapagliflozin for 14 days.

### Case 4

A 42 years lady with IgA nephritis for 20 years started CCPD because of hyperkalemia. The serum potassium was 7.2 meq/L, and she had a junctional rhythm on the electrocardiograph. The serum potassium decreased to 5.2 meq/L after PD. She also had legs pitting edema 4 + , and dapagliflozin 5 mg per day was prescribed. The ultrafiltration volume increased to 1267 ± 288 ml, and her leg pitting edema improved. The patient took dapagliflozin for 14 days.

## Discussion and conclusions

The kidney and the liver metabolize dapagliflozin. The glycemic efficacy of dapagliflozin is dependent on renal function, and efficacy is reduced in patients with impaired renal function [3]. Dapagliflozin overdose may lead to hyperkalemia, hyperphosphatemia, hypotension [4], acute kidney injury [5], and ketoacidosis [6]. These are the significant concerns in its use on dialysis patients. We started with a half-dose of dapagliflozin in all patients and monitored blood pressure, blood glucose, potassium, phosphate, and venous pH. We did not observe increased urine volume in PD patients, possibly because the effect of glucose diuresis was decreased in patients with chronic kidney disease. Case 4 did not have diabetes, and her blood glucose was monitored four times a day for three days. We did not find a hypoglycemia episode. The increased ultrafiltration may be explained by the decreased glucose resorption of the peritoneum [1, 2]. The glucose concentration was maintained in the PD fluid and increased osmotic water transport. The reported cases did not have ultrafiltration failure because they had 500-1000 ml of daily hyperfiltration. Most patients took dapagliflozin for 14 to 28 days, and we stopped dapagliflozin when patients were in euvolemic status. Although we reported an increase in ultrafiltration after dapagliflozin in four new PD cases, more studies were needed to support the clinical application and safety of dapagliflozin in PD patients. Dapagliflozin in PD patients remained limited by its off-label use because dapagliflozin is not indicated in end-stage renal disease patients.

A peritoneal equilibration test was suggested in a stable PD treatment of one to three months of PD. A 4-h fast peritoneal equilibration test was performed in the four cases to access the peritoneal function. All patients had high peritoneal equilibration tests on PD catheter insertion. Inflammation caused by the high concentrated glucose in PD fluid may explain the high peritoneal equilibration tests in naïve PD patients. Peritoneal equilibration tests may provide little information on the ultrafiltration on incident PD patients.

## Conclusions

SGLT-2 inhibitors may increase ultrafiltration in incident PD patients to achieve a euvolemic status. More studies are needed to support the applications of SGLT-2 inhibitors in PD patients with or without diabetes and their long-term effect on peritoneal function.

## Data Availability

The data supporting the findings of this study are available within the article.

## Abbreviations

CKD: Chronic kidney disease
CCPD: Continuous cycling peritoneal dialysis
PET: Peritoneal equilibration test
PD: Peritoneal dialysis
SGLT-2: Sodium glucose cotransporter-2

## References

[1] Zhou Y, Fan J, Zheng C, Yin P, Wu H, Li X, Luo N, Yu X, Chen C (2019). SGLT-2 inhibitors reduce glucose absorption from peritoneal dialysis solution by suppressing the activity of SGLT-2. Biomed Pharmacother.

[2] Balzer MS, Rong S, Nordlohne J, Zemtsovski JD, Schmidt S, Stapel B, Bartosova M, von Vietinghoff S, Haller H, Schmitt CP (2020). SGLT2 Inhibition by Intraperitoneal Dapagliflozin Mitigates Peritoneal Fibrosis and Ultrafiltration Failure in a Mouse Model of Chronic Peritoneal Exposure to High-Glucose Dialysate. Biomolecules.

[3] Komoroski B, Vachharajani N, Boulton D, Kornhauser D, Geraldes M, Li L, Pfister M (2009). Dapagliflozin, a novel SGLT2 inhibitor, induces dose-dependent glucosuria in healthy subjects. Clin Pharmacol Ther.

[4] Davies M, Chatterjee S, Khunti K (2016). The treatment of type 2 diabetes in the presence of renal impairment: what we should know about newer therapies. Clin Pharmacol.

[5] Pleros C, Stamataki E, Papadaki A, Damianakis N, Poulidaki R, Gakiopoulou C, Tzanakis I (2018). Dapagliflozin as a cause of acute tubular necrosis with heavy consequences: a case report. CEN Case Rep.

[6] Lee IH, Ahn DJ (2020). Dapagliflozin-associated euglycemic diabetic ketoacidosis in a patient with type 2 diabetes mellitus: A case report. Medicine (Baltimore).

